# Supplementary material for: Proton Treatment Suppresses Exosome Production in Head and Neck Squamous Cell Carcinoma
Source: Cancers (Basel). 2024 Feb 29;16(5):1008. doi: 10.3390/cancers16051008 (PMC10931005; doi:10.3390/cancers16051008)
Supplement: Supplementary file 1 [file cancers-16-01008-s001.zip › cancers-2865106-supplementary.pdf]

Supplementary Material:

Supplementary Methods:

Exosome western blot:

The abundance of EV (extracellular vesicles)-associated tetraspanin CD9 and extracellular vesicle (EV) binding protein TSG101 was determined by Western blot using the method described by Langevin et al., 2017 (25). 80 x 10^8 particles (as determined by NTA) EVs isolated from Cal27 cells by ultracentrifugation were mixed with 4x Laemmli SDS sample buffer (ThermoFisher) and 10x NuPAGE sample reducing agent (ThermoFisher) and heated at 95°C for 7 minutes. The denatured samples were then loaded onto each lane of a 4-12% Bis-Tris gels in an X Cell SureLock™ Gel System with MOPS SDS Running Buffer with added NuPAGE Antioxidant (all from ThermoFisher). Precision Plus Protein™ Kaleidoscope™ Prestained Protein Standards (BioRad) were loaded as a reference for protein size. Gels were run at 200V constant voltage and proteins were subsequently transferred onto polyvinylidene difluoride (PVDF) membrane (ThermoFisher) using Step 1- Transfer buffer at 1.3A constant current on a Pierce Power Station (ThermoFisher). The membranes were blocked with 5% BSA in 1x tris-buffered saline containing Tween-20 (TBS-T, MilliporeSigma) for one hour at room temperature and subsequently incubated overnight at 4°C with primary antibodies against CD9 (SBI) or TSG101 (Abcam) at 1:1000 dilution in 5% BSA in TBS-T. The blots were thoroughly washed with TBS-T, and incubated with either goat anti-rabbit HRP secondary antibody (SBI, for CD9) at 1:10000 dilution in 5% BSA in TBS-T for one hour, or antibody Goat anti-Rabbit IgG H&L (Abcam, for TSG101) at 1:2000 dilution in 5% BSA in TBS-T for two hours. The blots were visualized on a ChemiDoc XRS (Bio Rad, Hercules, CA) using Super Signal™ West Femto Maximum Sensitivity Substrate (ThermoFisher) as per the manufacturer's protocol.

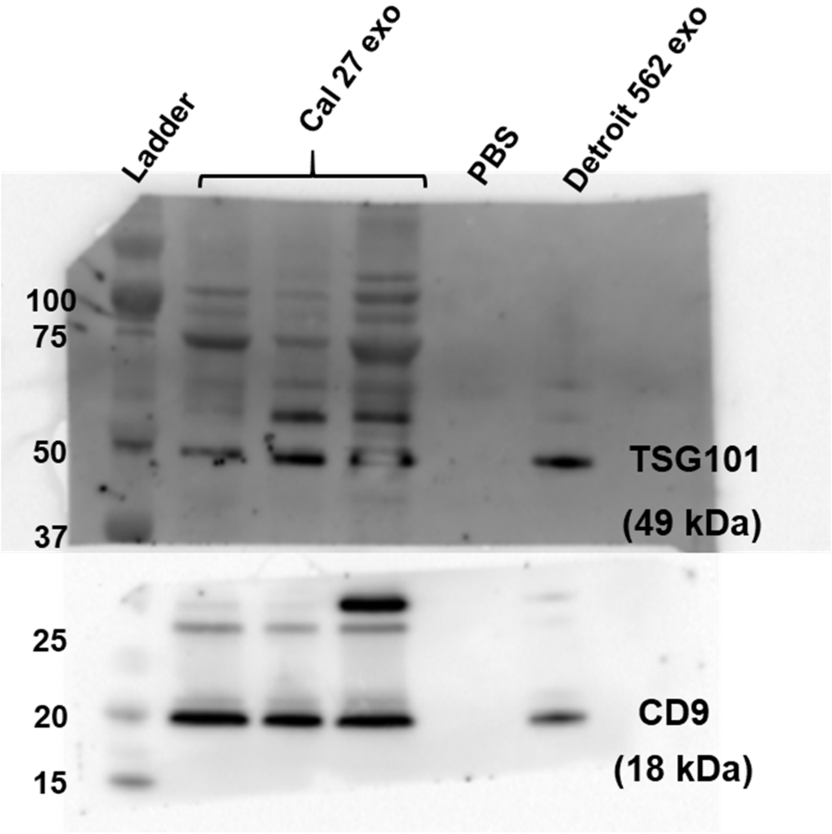

Figure S1. Western blotting to detect tetraspanins in extracellular vesicles isolated from cell supernatants of HNSCC cell lines.

Western blot analysis of protein expression of exosome-associated tetraspanin CD9 and exosome binding protein TSG101 for extracellular vesicles isolated by ultracentrifugation from conditioned media of the HNSCC cell lines Cal27 and Detroit562. No template controls (phosphate buffered saline (PBS) only) were included as negative controls. The Cal27-derived exosome samples run in this blot were obtained from three separate isolations. Shown here is a representative western blot, and this entire experiment was repeated twice.
